# A Calcineurin Inhibitor as an Enhancer of *Candida* Susceptibility to Anidulafungin

**DOI:** 10.1021/acsomega.5c10111

**Published:** 2026-01-29

**Authors:** Irene Rovira, Edurne Montemayor, Sandra Gil-Alonso, Katherine Miranda-Cadena, Ferran Sanchez-Reus, Guillermo Quindós, Elena Eraso, Nerea Jauregizar

**Affiliations:** † Department of Pharmacology, Faculty of Medicine and Nursing, 58349 University of the Basque Country (UPV/EHU), Barrio Sarriena, s/n, 48940 Bilbao, Spain; ‡ Department of Immunology, Microbiology and Parasitology, Faculty of Medicine and Nursing, 58349 University of the Basque Country (UPV/EHU), Barrio Sarriena, s/n, 48940 Bilbao, Spain; § Department of Immunology, Microbiology and Parasitology, Faculty of Science and Technology, 58349 University of the Basque Country (UPV/EHU), Barrio Sarriena, s/n, 48940 Bilbao, Spain; ∥ Servei de Microbiologia, 16689 Hospital de la Santa Creu i Sant Pau, C/San Quintín 89, 08024 Barcelona, Spain; ⊥ Instituto de Investigación Sanitaria Biobizkaia, 48903 Barakaldo, Spain

## Abstract

The increase in invasive candidiasis caused by emerging *Candida* species presents a significant medical challenge due to their resistance to conventional antifungal treatments. Exploring novel therapeutic strategies, such as drug repurposing, is therefore essential. Tacrolimus, an immunosuppressive calcineurin inhibitor, shows potential antifungal effects. This study investigated the in vitro effect of combining tacrolimus and echinocandin anidulafungin using the checkerboard method against 60 clinical *Candida* isolates, including *Candida albicans*, *Candida auris*, *Candida glabrata*, and *Candida parapsilosis*. Time-kill assays against *C. parapsilosis* clinical isolates with *FKS1* gene mutations were conducted. Checkerboard data revealed that tacrolimus reduced anidulafungin MIC values: a synergistic activity was observed against 90% of the isolates studied, with a particularly notable effect against multidrug-resistant *C. auris*, showing 100% synergy. Positive outcomes were corroborated by time-kill assays. Hence, the addition of tacrolimus may enhance susceptibility to anidulafungin, as suggested by checkerboard and time-kill assays conducted against *C. parapsilosis*, including isolates harboring mutations in *FKS1*. These promising findings are a starting point for future studies on the antifungal properties of tacrolimus and related compounds.

## Graphical abstract

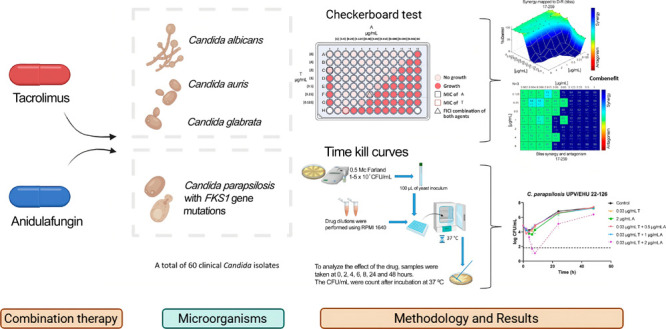

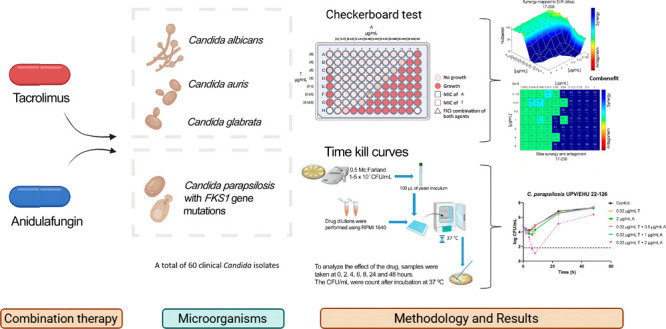

## Introduction

Invasive fungal infections have become an increasing problem worldwide with *Candida* species as the most common etiological agents, going hand in hand with the growing number of immunocompromised patients.^1^ *Candida albicans* has been the most frequent isolated species. However, there has been a gradual etiological change and the incidence of species such as *Candida glabrata* (*Nakaseomyces glabratus*) and *Candida parapsilosis* have increased.^2,3^ This event has an important clinical impact, as both species are less susceptible to some antifungal drugs: *C. glabrata* is intrinsically less susceptible to azole antifungals, particularly to fluconazole,^4^ while *C. parapsilosis* has an intrinsic resistance to echinocandins due to the natural polymorphism in *FKS1* gene.^5^ Moreover, *Candida auris* (*Candidozyma auris*), an emerging *Candida* species that causes important nosocomial outbreaks globally since 2009, has been described as highly resistant to most antifungals available, and frequently multidrug-resistant.^6^

The increasing resistance mechanisms being developed by *Candida* species together with the limited antifungal tools available makes treatment of invasive candidiasis an important medical challenge. The development of antifungal drugs is restricted due to fungi being eukaryotes, so potential targets for these pathogens are also found in humans. The development of new molecules is a resource-consuming process in terms of time and cost, therefore, there is an increasing interest in treatment alternatives such as combination therapy or drug repurposing. Combination therapy sustains the idea that drugs with different mechanisms of action can produce synergistic effects improving the efficiency of the treatment and reducing both dosage and the occurrence of drug-resistance.^7^ Drug repurposing consists of identifying new therapeutic uses of approved drugs that differ from its original purpose. This strategy presents some advantages such as the assured safety, as the drug has already been in clinical use, and the reduction in development time and investment.^8^ On this approach, several studies showed that calcineurin inhibitors, such as cyclosporin A and tacrolimus have synergistic activity when combined with antifungals which results in growth inhibition of resistant isolates.^9–13^

Tacrolimus is a calcineurin inhibitor clinically used as an immunosuppressor in the prophylaxis of allograft transplant rejection of liver, kidney or heart.^13^ It binds to the cytosolic protein FKBP12, forming a complex that inhibits calcineurin in a specific and competitive way.^14^ Calcineurin is a phosphatase protein crucial for the response of cell stress in eukaryotes, including fungi. This protein is activated when forming a complex with calmodulin, a protein that senses abnormally increased intracellular Ca^2+^ concentrations.^15^ This complex activates virulence genes and proteins related to cell wall synthesis, cellular cycle regulation, thermotolerance, cation homeostasis and antifungal resistance.^16^ Calcineurin is an essential protein; therefore, its inhibition directly affects the viability of fungal cells, making it a potential antifungal target.

In this context, the aim of this study was to analyze the in vitro activity of tacrolimus alone and in combination with the echinocandin anidulafungin against four relevant species of *Candida* by checkerboard and time-kill combination method. The species selection criteria for this study were, on one hand, species commonly isolated in invasive candidiasis, and on the other hand, emerging and resistant species. Demonstrating the antifungal effect of tacrolimus could become the starting point for the research of modified molecules with similar antifungal activity but reduced immunosuppressive activity, in order to establish new therapeutic methods against invasive candidiasis.

## Results

### Checkerboard Assay

The MIC values against the 60 *Candida* isolates tested in the checkerboard assay are represented in Table 1. This summary table enables the comparison of the antifungal effect of anidulafungin and tacrolimus against the four different species of *Candida* studied.

**Table 1:** Comparison of MICs of Anidulafungin and Tacrolimus in Monotherapy or in Combination against 60 *Candida* Isolates^a^

		monotherapy		combination therapy					
species		anidulafungin	tacrolimus	anidulafungin	tacrolimus	Loewe theory		Bliss theory	
*Candida* spp. (60)	range	0.004 to ≥1	0.03 to ≥8	≤0.002 to ≥1	0.03–4	range FICI	0.03–1.01	range ΣSYN_ANT	–26.57 to 223.75
	GM	0.34	2.607	0.023	0.198				
	MM	≥1	≥8	≤0.002	0.125				
	MIC_50_	1	4	0.03	0.125				
	MIC_90_	≥1	≥8	0.5	1				
*C. albicans* (13)	range	0.004–0.03	≥8	≤0.002–0.004	0.125–4	range FICI	0.26–1.01	range ΣSYN_ANT	–6.714 to 118.56
	GM	0.014	≥8	0.0038	0.653				
	MM	0.008	≥8	0.004	1				
	MIC_50_	0.008	≥8	0.004	1				
	MIC_90_	0.03	≥8	0.004	2				
*C. auris* (12)	range	≥1	≥8	0.03–0.125	0.125–2	range FICI	0.03–0.13	range ΣSYN_ANT	103.43 to 223.75
	GM	≥1	≥8	0.068	0.297				
	MM	≥1	≥8	0.06	0.125				
	MIC_50_	≥1	≥8	0.06	0.125				
	MIC_90_	≥1	≥8	0.125	1				
*C. glabrata* (13)	range	0.03 to ≥ 1	0.125 to ≥2	≤0.002–0.125	0.03–2	range FICI	0.27–1.00	range ΣSYN_ANT	–26.57 to 54.56
	GM	0.15	1.798	0.023	0.136				
	MM	0.06	≥2	0.03	0.03				
	MIC_50_	0.06	≥2	0.03	0.06				
	MIC_90_	0.5	≥2	0.03	0.5				
*C. parapsilosis* (22)	range	0.06 to ≥1	0.06 to ≥2	≤0.002 to ≥1	0.03 to ≥2	range FICI	0.24–1.00	range ΣSYN_ANT	–23.90 to 133.69
	GM	1.412	0.413	0.037	0.098				
	MM	≥1	0.125	≤0.002	0.125				
	MIC_50_	≥1	0.25	0.25	0.06				
	MIC_90_	≥1	≥2	1	0.5				

a MM = modal MIC; GM = geometric mean MIC; MIC_50_ = MIC at which 50% of the isolates was inhibited; MIC_90_ = MIC at which 90% of the isolates was inhibited. SYN = synergy; range FICI = range of fractional inhibitory concentration index; and range ΣSYN_ANT = range of total sum of synergic and antagonistic interactions by the Bliss model.

#### C. albicans

The MIC values for anidulafungin against the 13 *C. albicans* isolates studied ranged from 0.004 to 0.03 μg/mL. The highest concentration of tacrolimus tested was unable to inhibit the growth of these isolates, hence the MIC was ≥8 μg/mL. Comparing the MIC values in monotherapy and in combination, a 3.7-fold decrease was observed in the geometric mean (GM) of the MIC of anidulafungin in combination (0.0038 μg/mL) in comparison with the MIC of anidulafungin alone (0.014 μg/mL). The GM for tacrolimus in combination (0.6527 μg/mL) was 24.5 times lower than in monotherapy (≥8 μg/mL). The FICI model revealed synergy against 10 of the isolates tested. The mean FICI (SD) was 0.45 (0.20), which is consistent with synergistic activity against *C. albicans*, and similar results were observed using the Bliss model, concluding a weak synergistic interaction in all but one isolate (Supplementary Table S1 and Figure S1). Overall, synergy between anidulafungin and tacrolimus was defined against 12 out of the 13 *C. albicans* isolates.

#### C. auris

The MIC values of anidulafungin and tacrolimus in monotherapy against the 12 isolates of *C. auris* were between ≥1 and ≥ 8 μg/mL, respectively. When both drugs were combined, a decrease in the MIC values of anidulafungin (MIC *A*_comb_ range: 0.03–0.125 μg/mL) and tacrolimus (MIC *T*_comb_ range: 0.125–2 μg/mL) was observed. The FICI values (0.03–0.13) indicated a synergistic interaction in all assays (Supplementary Table S2). The Bliss model confirmed a strong or moderate synergy between tacrolimus and anidulafungin against the 12 *C. auris* isolates studied. Strong synergy found in UPV/EHU 17–259 isolate is illustrated in Figure 1.

**Figure 1:**
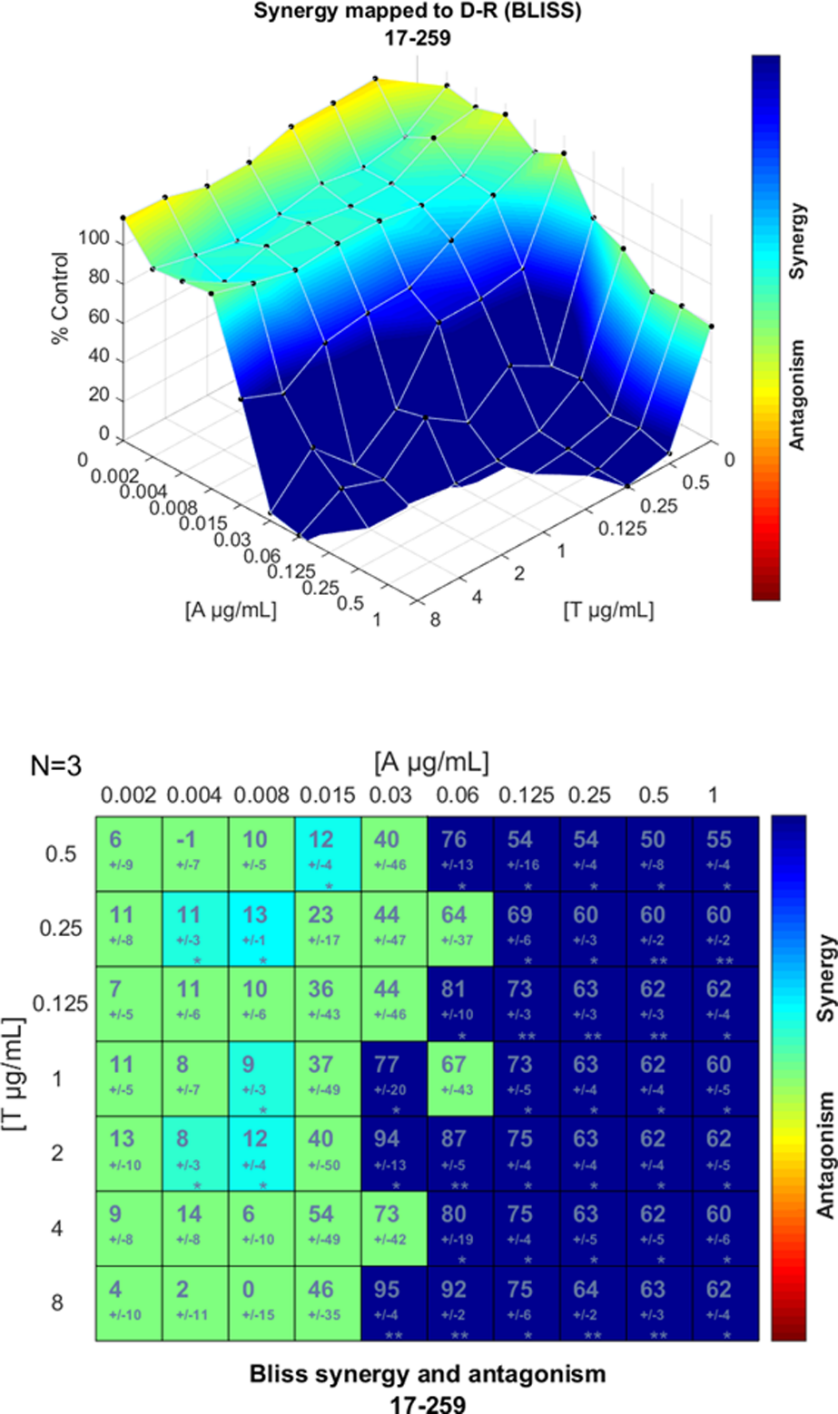
Synergy distribution determined by the Bliss interaction model for the combination of anidulafungin and tacrolimus against *C. auris* UPV/EHU 17-259. Top: synergy distribution mapped to the dose–response surface. Bottom: Matrix synergy plot with synergy scores for each combination.

#### C. glabrata

According to the EUCAST clinical breakpoints, four out of 13 isolates of *C. glabrata* studied were considered resistant. The MIC values for anidulafungin against the resistant isolates were 0.5–≥1 μg/mL, whereas against the susceptible isolates, the MIC ranged from 0.03 to 0.06 μg/mL. Tacrolimus in monotherapy showed some inhibition activity against three isolates (MIC 0.125 μg/mL), but in the remaining nine, the highest concentration used had no effect (MIC ≥ 2 μg/mL). When the combination of both drugs was tested, the MIC values against *C. glabrata* were significantly lower. A 6.45-fold decrease was observed when comparing the GM of MIC of anidulafungin alone (0.1504 μg/mL) and the MIC in combination (0.0233 μg/mL). For tacrolimus, the fold reduction in GM from MIC of tacrolimus in monotherapy (1.799 μg/mL) to MIC in combination (0.1365 μg/mL) was 13.18.

The combination of anidulafungin and tacrolimus against *C. glabrata* resulted in synergistic activity in seven out of the 13 isolates tested and an indifferent effect in the remaining six, as determined by FICI analysis. Although the mean FICI (SD) was 0.65 (0.24), indicating an overall indifferent interaction, synergistic activity was observed in more than half of the isolates evaluated. It was observed that against two resistant isolates, although the FICI value showed an indifferent interaction between the drugs, an addition of 0.125 μg/mL of tacrolimus was able to decrease the MIC value for anidulafungin from ≥1 μg/mL to as low as ≤0.002 μg/mL. The Bliss model confirmed the weak synergy against 76.9% of the isolates (Supplementary Table S3 and Figure S2).

#### C. parapsilosis

The MIC values for anidulafungin and tacrolimus in monotherapy against 22 isolates of *C. parapsilosis* ranged from 0.06 to ≥1 and 0.06 to ≥2 μg/mL, respectively. The combination of both drugs resulted in a 38.25-fold decrease in the MIC of anidulafungin (GM *A*_alone_ = 1.4116; GM *A*_comb_ = 0.0369 μg/mL) and 4.21-fold decrease in the MIC of tacrolimus (GM *T*_alone_ = 0.4132; GM *T*_comb_ = 0.0982 μg/mL). Therefore, the addition of tacrolimus reduced the concentration of antifungal necessary to inhibit the growth of *C. parapsilosis*. The FICI model indicated a synergistic interaction between anidulafungin and tacrolimus against 13 out of 22 isolates (59.1%), with a mean FICI of 0.41 among the isolates with synergistic results, while the Bliss model described synergy against 17 isolates (77.3%) (Supplementary Table S4 and Figure S3). Overall, synergy was found against 19 out of 22 *C. parapsilosis* isolates (86.4%) by either model, Loewe theory or Bliss theory. No significant difference was observed between the synergy found in WT isolates and isolates with mutations in *FKS1* gene.

As shown in Table 1, the highest synergistic interactions between anidulafungin and tacrolimus were described against *C. auris*, followed by *C. albicans*, *C. parapsilosis*, and last, *C. glabrata*. The overall synergy rate against all 60 isolates was 90%, demonstrating a positive interaction of both drugs.

### Time-Kill Curves

Time-kill curves for anidulafungin and tacrolimus, in monotherapy and in combination, against four isolates of *C. parapsilosis* are illustrated in Figure 2. Neither of the drugs tested achieved significant antifungal activity by themselves, as observed on the plots, where no difference is noticeable between control curves and 2 μg/mL of anidulafungin or 0.03 μg/mL of tacrolimus. Combinations of 0.03 μg/mL of tacrolimus and 1 or 0.5 μg/mL of anidulafungin also resulted ineffective.

**Figure 2:**
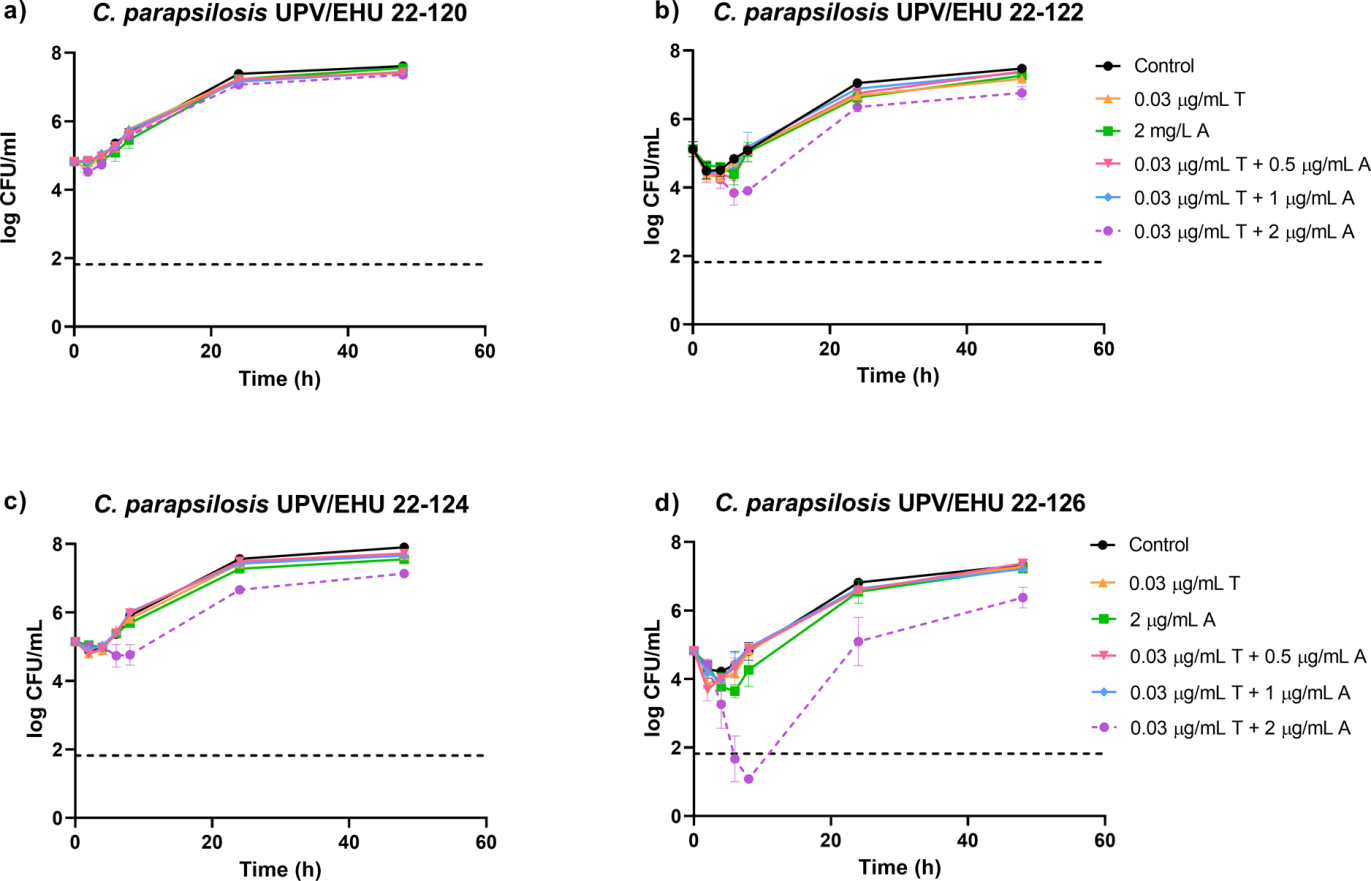
(a–d) Time-kill curves for anidulafungin (A) and tacrolimus (T) in monotherapy and in combination against four *C. parapsilosis* isolates: one wild-type, *C. parapsilosis* UPV/EHU 22-120, and three with mutations in the *FKS1* gene, *C. parapsilosis* UPV/EHU 22-122, *C. parapsilosis* UPV/EHU 22-124, and *C. parapsilosis* UPV/EHU 22-126. Each data point represents the mean result ± standard deviation (error bars) of all replicates.

While the combination of 0.03 μg/mL of tacrolimus and 2 μg/mL of anidulafungin showed no effect against the WT isolate UPV/EHU 22–120, some antifungal activity was observed against the clinical isolates with a mutation in the *FKS1* gene. Although the activity was not fungicidal according to the established definition (growth reduction ≥ 3 log_10_ CFU/mL from the initial inoculum) against the *C. parapsilosis* UPV/EHU 22-122 and *C. parapsilosis* UPV/EHU 22-124 isolates, the time-kill curve of the combination was slightly inferior to the control curve. No synergistic interaction was observed between tacrolimus and anidulafungin against these isolates.

As shown in Figure 2d, the combination of 0.03 μg/mL of tacrolimus and 2 μg/mL of anidulafungin against *C. parapsilosis* UPV/EHU 22-126 generated significant improvement in the fungistatic activity. Fungicidal activity was achieved at 6 and 8 h, with a growth reduction from the initial inoculum of 3.15 and 3.19 log_10_ CFU/mL, respectively. Although the results show some regrowth after 8 h, the combination curve remains lower at 24 and 48 h in comparison to the control curve. This combination of anidulafungin and tacrolimus could be defined as synergistic, as *a* > 2 log_10_ CFU/mL difference in the fungal count reduction was observed at 6 and 8 h between the drug with the greater effect in monotherapy, anidulafungin, and its corresponding combination.

## Discussion

The increasing incidence of invasive candidiasis caused by species of *Candida* with reduced susceptibility to antifungal drugs has made it essential to search for alternative therapeutic strategies.

Recent studies show that some nonantifungal drugs, such calcineurin inhibitors that act as immunosuppressant agents, have antifungal activity, as calcineurin is an essential protein for fungal survival.^9,10,17–20^ Calcineurin inhibitors in monotherapy have different antifungal effects depending on the fungal species. While these inhibitors have been proven as effective against *Aspergillus fumigatus*,^11^ their activity against *Saccharomyces cerevisiae*, *C. albicans*, *C. glabrata*, and *C. auris* was scarce.^9,10,21–23^ In contrast, inhibitory effect of tacrolimus and cyclosporine A, calcineurin inhibitors, on *C. parapsilosis* growth has previously been reported with MIC values of 0.125–0.5 and 6.25 μg/mL, respectively.^24,25^ The results of the present study confirmed the inefficacy of tacrolimus in monotherapy against *C. albicans* and *C. auris* (MIC ≥ 8 μg/mL). Interestingly, our results indicated that tacrolimus exhibited activity against three out of four *C. glabrata* isolates classified as resistant to anidulafungin according the EUCAST breakpoints, with a MIC value of 0.125 μg/mL. Moreover, the current study confirmed the inherent activity of tacrolimus against *C. parapsilosis* (GM = 0.413 μg/mL).

In the past decade, promising results in the treatment of fungal infections have been described with the combination of calcineurin inhibitors and antifungal drugs. In the present study, the in vitro interaction of tacrolimus and anidulafungin was investigated against 60 clinical *Candida* isolates of four different species of *Candida* (*C. albicans*, *C. auris*, *C. glabrata*, and *C. parapsilosis*). These combinations of antifungal drugs and calcineurin inhibitors has already been proven effective against *C. albicans*,^10,11,19,23,26,27^ *C. glabrata* ^9,20,21,23^ and *C. parapsilosis*.^23,24^ However, very few studies have investigated the effect of combination therapy with calcineurin inhibitors and echinocandins against the highly resistant *C. auris* species. A recent study demonstrated an increased susceptibility of *C. auris* to echinocandins when combined with tacrolimus.^28^ In contrast, the study by Khodavaisy et al. reported no synergism against five *C. auris* isolates that were susceptible to anidulafungin.^23^

The current study confirms the synergistic interaction between calcineurin inhibitors and antifungal drugs, as synergy was observed by either of the two models against 54 of the 60 isolates tested (90%), demonstrating a positive interaction of both drugs. This result is remarkable due to the large amount of antifungal susceptible and resistant isolates from different *Candida* species studied, including some clinical isolates of *C. parapsilosis* with mutations in the *FKS1* gene that may be associated with reduced susceptibility to echinocandins. The combination of tacrolimus with anidulafungin may be promising in the treatment of different candidiasis. Interestingly, the most remarkable outcome corresponded to the multidrug-resistant species *C. auris*, in which synergistic activity was detected against all isolates tested.

Echinocandins inhibit 1,3- β-d-glucan synthesis, leading to cell wall integrity stress. This cell wall perturbation triggers compensatory mechanisms, such as Ca^2^^+^/calcineurin-dependent signaling in order to maintain cell wall adaptation and viability. Inhibition of the calcineurin pathway can therefore impairs this compensatory response, providing a plausible explanation for the synergistic interaction observed between echinocandins and calcineurin inhibitors, including tacrolimus.^29^ The essential role of calcineurin in stress adaptation and fitness in *C. glabrata* has been demonstrated through genome-wide in vitro analysis^30^ Furthermore, a recent study explored the complex contribution of the calcineurin signaling pathway to *C. auris* pathogenicity, both in vitro and in animal models. The authors reported that deletion of the calcineurin complex compromises cell wall integrity, increasing susceptibility to echinocandins and accounting for the observed synergy these agents are combined against *C. auris*.^28^

It has been described that in most *Candida* species the reduced susceptibility to echinocandins is associated with a natural polymorphism in two hotspot (HS) regions of the *FKS1* and *FKS2* genes.^31,32^ On this approach, Martí-Carrizosa et al.^33^ studied the susceptibility of 11 *C. parapsilosis* isolates to echinocandins; these isolates harbored mutations outside the HS regions of *FKS1*, and resistance was described in only two (MIC range 1–8 μg/mL). Similar results were obtained in the present work, including some of the isolates from the Martí-Carrizosa et al.^33^ study (MIC ≥ 1 μg/mL). In the checkerboard method, synergy between anidulafungin and tacrolimus was found against 88.9% of *C. parapsilosis* isolates carrying the described mutations, with anidulafungin MICs in combination decreasing to as low as ≤0.002 μg/mL. This suggests that although the mutations were not located within HS regions, the addition of tacrolimus could reverse echinocandin resistance as previously reported.^20,22,24,27,34^ Furthermore, the results of time-kill assays showed that the combination of tacrolimus and anidulafungin had a greater effect against the isolates with mutations than against the WT isolate, supporting this hypothesis.

Katiyar et al.^34^ described that the function of *FKS* genes differs between species of *Candida: FKS1* and *FKS2* genes are fully redundant in *C. glabrata* and this redundancy is lacking in *C. albicans*, as fks1Δ isolates are nonviable. These authors suggested that the redundancy occurring in *C. glabrata* attenuated the impact of the resistance-conferring mutations.^34^ This hypothesis correlates with Martí-Carrizosa et al.^33^ and our current results, where different susceptibility models described isolates with mutation in non-HS regions of *FKS1* as nonresistant against echinocandins. Furthermore, Katiyar et al.^34^ affirms that calcineurin inhibitors have an antifungal effect on *FKS1* mutants but not in *FKS2* mutants, suggesting that calcineurin is specifically required for *FKS2* activity in *C. glabrata*. The MIC_50_ of tacrolimus in monotherapy of 0.25 μg/mL against the isolates with mutations in *FKS1* tested in the current study could correlate to this hypothesis, showing that tacrolimus alone has some antifungal effects against these isolates.

The in vitro interaction between drugs was assessed using the time-kill method, which enables the evaluation of drug pharmacokinetics and checkerboard assays.^35^ Checkerboard results were analyzed by the FICI model, a simple method with associated variability,^36^ and response surface analysis by the Bliss model, which includes all the generated data reducing the variability.^37^ As the behavior of calcineurin inhibitors could be different in vivo and in vitro, further studies on the in vivo activity of the combination of anidulafungin and tacrolimus are required. Chen et al.^38^ proved that a synergistic interaction was found in vitro between posaconazole and tacrolimus against *C. albicans*; however, in the murine model, the addition of tacrolimus significantly reduced the survival rate. It is suggested that its immunosuppressive activity could be aggravating these infections. On this approach, Lee et al.^39^ developed four tacrolimus analogues and compared their cytotoxicity, immunosuppressive activity, and antifungal activities against three major human fungal pathogens, *Cryptococcus neoformans*, *C. albicans*, and *A. fumigatus*. One of these analogues exhibited a reduced immunosuppressive activity compared to tacrolimus retaining a significant antifungal activity, which makes it a promising therapeutic alternative against candidiasis. The combination of fluconazole and a less toxic analogue of tacrolimus against *Cryptococcus neoformans* in a murine model, finding an increase in the survival rate. While other research has focused on the combination of azole antifungals with tacrolimus, such coadministration requires caution due to the well-documented interaction between azoles and cytochrome P450 enzymes, particularly CYP3A4. In contrast, echinocandins like anidulafungin are not significant inhibitors of CYP3A4, suggesting a potentially safer pharmacological profile when used with immunosuppressive agents. These considerations highlight the importance of further investigating echinocandin–tacrolimus combinations.

## Conclusions

While tacrolimus alone at the tested concentrations presented poor antifungal activity against *Candida*, its combination with anidulafungin was found to be potentially active and synergistic in 90% of the studied isolates. It exhibited variable effects depending on the species: *C. auris* resulted as the most susceptible, followed by *C. albicans*, *C. parapsilosis*, and last, *C. glabrata*. Furthermore, our results from checkerboard and time-kill assays on *C. parapsilosis* with mutations in *FKS1* gene suggest that the addition of tacrolimus could increase the susceptibility of these isolates to anidulafungin. The results obtained constitute a starting point to consider for future studies on the antifungal activity of tacrolimus.

## Methods

### Fungal Isolates and Drugs

In order to test the in vitro susceptibility to anidulafungin and tacrolimus, a total of 60 clinical *Candida* isolates belonging to *C. albicans* (*n* = 13), *C. auris* (*n* = 12), *C. glabrata* (*n* = 13), and *C. parapsilosis* (*n* = 22) were selected. Nine of the clinical isolates of *C. parapsilosis* were obtained from the Hospital de la Santa Creu i Sant Pau (Barcelona, Spain) and harbored mutations outside the two HS regions of the *FKS1* gene in addition to the previously described naturally occurring P660A polymorphism in the HS1 region of the *FKS1* gene. The nomenclature and anatomic origin of the isolates are described in Table 2. The antifungal drug anidulafungin (Pfizer, SLU, Spain) and the immunosuppressive drug tacrolimus (Sigma-Aldrich, Spain, 98% HPLC purity) were used in this study. These products were obtained in powder form and they were dissolved in dimethyl sulfoxide to reach stock concentrations of 3200 μg/mL. All stock solutions were stored at −80 °C until use.

**Table 2:** List of the *Candida* Clinical Isolates Studied^a^

species	isolate UPV/EHU	clinical specimen	original code/amino acid change(s) in **Fks1**
*Candida albicans*	17–009, 17–068, 17–071	blood	
	17–072, 17–073, 17–074		
	17–190, 17–192, 17–296		
	17–299, 17–348, 17–349		
	18–022		
*Candida auris*	17–213	blood	CJ110
	17–257	blood	CJ94
	17–259	blood	CJ97
	17–263	blood	CJ99
	17–265	blood	CJ100
	17–267	blood	CJ102
	18–029	blood	JMRC: NRZ1 101
	17–276	oropharyngeal	CR312
	17–279	oropharyngeal	CR309
	17–280	urine	CR176
	17–281	urine	CR201
	17–285	urine	CR220
*Candida glabrata*	03–273, 17–013, 17–077	blood	
	17–078, 17–164, 17–173		
	17–181, 17–236, 17–237		
	17–238, 17–242, 17–297		
	22–119	blood	1176 LL
*Candida parapsilosis* WT	17–031, 17–037, 17–109	blood	
	17–112, 17–113, 17–114		
	17–250, 17–251, 17–254		
	17–311, 17–327, 17–329		
	22–120	blood	1161 LL WT
*Candida parapsilosis FKS1* mut	22–121	blood	1064 LL/M1328I
	22–122	blood	1031 LL/S745S/L
	22–123	blood	928 LL/V595I
	22–124	blood	831 LL/S745L
	22–125	blood	713 LL/S74SS/L
	22–126	blood	681 LL/A1422G
	22–127	blood	544 LL/S745S/L
	22–128	blood	474 LL/M1328I
	22–129	blood	462 LL/S745S/L

a WT: wild-type. *FKS1* mut: isolates with a mutation in the *FKS1* gene donated from Servei de Microbiologia, Hospital de la Santa Creu i Sant Pau, Barcelona, Spain (Martí-Carrizosa et al.). UPV/EHU: Collection of Universidad del País Vasco/Euskal Herriko Unibertsitatea. *C. auris* isolates were donated from Hospital Universitario y Politécnico La Fe, Valencia, Spain, and one isolate (18-029) from University Hospital Cologne, Cologne, Germany.

### Antifungal Susceptibility Testing

Antifungal susceptibility was studied by the determination of minimal inhibitory concentration (MIC) following EUCAST guidelines.^40^ MIC values are defined as the lowest drug concentration that produces a 50% inhibition of the growth of a microorganism. In order to determine this value, a gradient of concentrations was tested against an inoculum of 0.5–2.5 × 10^5^ colony forming units per mL (CFU/mL) of *Candida* isolates. The fungal suspensions were diluted with RPMI-1640 supplemented with l-glutamine and buffered at pH 7 with 0.165 M of 3-(*N*-morpholino) propanesulfonic acid (MOPS, Sigma-Aldrich). *C. albicans* ATCC 64550 and *C. parapsilosis* ATCC 22019 were used as quality controls. Based on EUCAST Clinical breakpoints microorganisms were categorized as clinically susceptible or resistant.^41^

### Checkerboard Assay

The potential interaction between anidulafungin and tacrolimus was evaluated based on EUCAST guidelines by a checkerboard assay.^42^ The experiment was carried out in 96-well flat-bottom microtiter plates with RPMI medium and a gradient of concentrations of the two drugs that was different for each *Candida* species. The concentrations of anidulafungin ranged from 0.002 to 1 μg/mL and were added into columns. Against *C. auris* and *C. albicans*, the range of tacrolimus concentrations was 0.125–8 μg/mL, while a range of 0.03–2 μg/mL was used against *C. glabrata* and *C. parapsilosis*. Tacrolimus was added to rows. Growth control and sterility control wells were also included in the plate. On the day of the experiment, *Candida* isolates, previously incubated at 37 °C overnight, were added to the microtiter plates to obtain an initial inoculum of 0.5–2.5 × 10^5^ CFU/mL. The plates were incubated at 37 °C for 24 or 48 h and the absorbance was measured on a spectrophotometer (Infinite F50, Tecan, Switzerland), at a wavelength of 450 nm. With these data, the MIC of each drug alone and in combination were determined. Experiments were conducted by triplicate on different days.

#### Data Analysis

The in vitro interaction of the drug combination was analyzed by the Fractional Inhibitory Concentration Index (FICI) model and the Bliss independence model. The nonparametric FICI model was expressed using eq 1:

$$\mathrm{FICI}=\frac{{{\mathrm{MIC}}_{A}}^{\mathrm{comb}}\;}{{{\mathrm{MIC}}_{A}}^{\mathrm{alone}}}+\frac{{{\mathrm{MIC}}_{T}}^{\mathrm{comb}}}{{{\mathrm{MIC}}_{T}}^{\mathrm{alone}}}$$ 1

where MIC_A_^alone^ and MIC_T_^alone^ were the MICs of drugs anidulafungin and tacrolimus in monotherapy and MIC_A_^comb^ and MIC_T_^comb^ when used in combination. Three different drug interactions can be defined depending on the FICI value: synergistic if FICI ≤ 0.5, no interaction if 0.5 > FICI ≤ 4 and antagonistic if FICI > 4.^43^ Additionally, the checkerboard results were analyzed by response surface analysis, using Combenefit software (University of Cambridge, UK) and following the Bliss independence theory. Combenefit generates a reference surface that is evaluated from the dose–response curves of each of the two combined drugs and a matrix plot where the synergism or antagonism score for each combination and its statistical significance are represented. The sums of the percentages of all statistically significant synergistic and antagonistic interactions were calculated (ΣSYN_ANT) in order to classify the interaction as weak (<100%), moderate (100–200%) or strong (>200%).^36^ The interaction between anidulafungin and tacrolimus was considered significant if it was confirmed with at least one model, either FICI or Bliss model.^21^

### Time-Kill Assay

Static time-kill curve experiments were carried out to further investigate the pharmacokinetics of the interaction between tacrolimus and anidulafungin.^44^ These assays were conducted on four isolates of *C. parapsilosis*: one wild-type (WT) and three clinical isolates with a mutation in the *FKS1* gene. In order to assess the interaction between the two drugs, the concentrations used in combination were also studied in monotherapy (2 μg/mL for anidulafungin and 0.03 μg/mL for tacrolimus). Microtiter plates were used to display the drugs in combination or alone. RPMI medium was used to prepare the concentrations of drugs (0.5, 1, 2 μg/mL of anidulafungin and 0.03 μg/mL of tacrolimus) and for growth control. *C. parapsilosis* isolates were incubated 24 h before the start of the experiment at 37 °C and the starting inoculum concentration was (1–5) × 10^5^ CFU/mL. In order to count viable cells, samples were taken at 0, 2, 4, 6, 8, 24, and 48 h and spread in triplicate onto Sabouraud dextrose agar. CFU/mL were counted 48 h afterward. Each assay was performed in duplicate on different weeks.

#### Data Analysis

The time-kill data were analyzed using the GraphPad Prism 8 (USA) software, and a graphic representation of log CFU/mL versus time was created for the drugs in monotherapy and in combination. The possible effect of the drugs was defined as fungistatic if a reduction <3 log CFU/mL from the initial inoculum occurred, or fungicidal if the reduction was ≥3 log CFU/mL.^45^ Moreover, the nature of the interaction between two drugs can be defined as synergistic when the difference in fungal count reduction between the drug with greater effect in monotherapy and its corresponding combination was ≥2 log CFU/mL. Indifference was described when the reduction was <2 log CFU/mL and antagonism, if an increase of ≥2 log CFU/mL in comparison with the most effective drug occurred.^44^

## Supplementary Material



## Notes

Tables S1–S4: in vitro interaction between anidulafungin and tacrolimus against *C. albicans*, *C. auris*, *C. glabrata*, and *C. parapsilosis*; and Figures S1–S3: synergy distribution determined by the Bliss interaction model for the combination of anidulafungin and tacrolimus against *C. albicans*, *C. glabrata*, and *C. parapsilosis* isolates (PDF)
