# Supplementary material for: A Calcineurin Inhibitor as an Enhancer of Candida Susceptibility to Anidulafungin
Source: ACS Omega. 2026 Jan 29;11(5):8315–22. doi: 10.1021/acsomega.5c10111 (PMC12902964; doi:10.1021/acsomega.5c10111)
Supplement: Supplementary file 1 [file ao5c10111_si_001.pdf]

Supporting Information

A calcineurin inhibitor as an enhancer of *Candida* susceptibility to anidulafungin

Irene Rovira[a], Edurne Montemayor[b], Sandra Gil-Alonso[c], Katherine Miranda-Cadena[b], Ferran Sanchez-Reus[d], Guillermo Quindós[b,e], Elena Eraso[b] and Nerea Jauregizar[a,e*]

[a]Department of Pharmacology, Faculty of Medicine and Nursing, University of the Basque Country (EHU), Barrio Sarriena, s/n, 48940, Bilbao, Spain

[b]Department of Immunology, Microbiology and Parasitology, Faculty of Medicine and Nursing, University of the Basque Country (EHU), Barrio Sarriena, s/n, 48940, Bilbao, Spain

[c]Department of Immunology, Microbiology and Parasitology, Faculty of Science and Technology, University of the Basque Country (EHU), Barrio Sarriena, s/n, 48940, Bilbao, Spain

[d]Servei de Microbiologia, Hospital de la Santa Creu i Sant Pau, C/ San Quintín 89, 08024, Barcelona, Spain

[e]Instituto de Investigación Sanitaria Biobizkaia, 48903, Barakaldo, Spain

* Corresponding author: Nerea Jauregizar, nerea.jauregizar@ehu.eus.

Table S1. *In vitro* interaction between anidulafungin and tacrolimus against *C. albicans*, using the non-parametric FICI model (Loewe) and the parametric Bliss model (ΣSYN_ANT).

C. albicans isolate	R/S	Loewe theory						Bliss theory	
		MIC A_{alone}	MIC A_{comb}	MIC T_{alone}	MIC T_{comb}	FICI	INT	ΣSYN_ ANT	INT
**UPV/EHU 17-009**	R	1	0.5	≥ 8	0.125	**0.50**	SYN	**23.98**	SYN weak
**UPV/EHU 17-068**	S	0.008	0.004	≥ 8	0.25	**0.50**	SYN	**34.41**	SYN weak
**UPV/EHU 17-071**	S	0.015	0.004	≥ 8	1	**0.33**	SYN	**50.42**	SYN weak
**UPV/EHU 17-072**	S	0.004	0.004	≥ 8	0.125	**1.01**	IND	**-6.714**	ANT weak
**UPV/EHU 17-073**	S	0.004	≤ 0.002	≥ 8	1	**0.56**	IND	**8.618**	SYN weak
**UPV/EHU 17-074**	S	0.008	≤ 0.002	≥ 8	4	**0.50**	SYN	**53.21**	SYN weak
**UPV/EHU 17-190**	S	0.008	≤ 0.002	≥ 8	2	**0.38**	SYN	**42.18**	SYN weak
**UPV/EHU 17-192**	S	0.008	≤ 0.002	≥ 8	1	**0.31**	SYN	**53.78**	SYN weak
**UPV/EHU 17-296**	S	0.03	0.004	≥ 8	2	**0.26**	SYN	**118.56**	SYN mod
**UPV/EHU 17-299**	S	0.008	0.004	≥ 8	1	**0.56**	IND	**32.29**	SYN weak
**UPV/EHU 17-348**	S	0.015	0.004	≥ 8	1	**0.33**	SYN	**66.46**	SYN weak
**UPV/EHU 17-349**	S	0.015	0.004	≥ 8	0.5	**0.30**	SYN	**58.11**	SYN weak
**UPV/EHU 18-022**	S	0.015	0.004	≥ 8	1	**0.33**	SYN	**67.55**	SYN weak

S: Susceptible (≤ 0.03 µg/mL); R: Resistant (> 0.03 µg/mL), following EUCAST Clinical breakpoints for anidulafungin [18]. A: Anidulafungin. T: Tacrolimus. FICI: Fractional Inhibitory Concentration Index. INT: Interpretation. ΣSYN_ANT: total sum of synergic and antagonistic interactions by Bliss model. SYN: synergy; IND: indifference; ANT: antagonism; SYN mod: moderate synergy.

Table S2. *In vitro* interaction between anidulafungin and tacrolimus against *C. auris*, by the non-parametric FICI model (Loewe) and the parametric Bliss model (ΣSYN_ANT).

C. auris isolate	R/S	Loewe theory						Bliss theory	
		MIC A_{alone}	MIC A_{comb}	MIC T_{alone}	MIC T_{comb}	FICI	INT	ΣSYN_ ANT	INT
**UPV/EHU 17-213**	N.A.	≥ 1	0.06	≥ 8	2	**0.09**	SYN	**179.77**	SYN mod
**UPV/EHU 17-257**	N.A.	≥ 1	0.06	≥ 8	0.125	**0.04**	SYN	**188.23**	SYN mod
**UPV/EHU 17-259**	N.A.	≥ 1	0.06	≥ 8	0.125	**0.04**	SYN	**223.75**	SYN strong
**UPV/EHU 17-263**	N.A.	≥ 1	0.125	≥ 8	0.5	**0.09**	SYN	**171.14**	SYN mod
**UPV/EHU 17-265**	N.A.	≥ 1	0.125	≥ 8	0.125	**0.07**	SYN	**173.67**	SYN mod
**UPV/EHU 17-267**	N.A.	≥ 1	0.06	≥ 8	0.5	**0.06**	SYN	**200.30**	SYN strong
**UPV/EHU 17-276**	N.A.	≥ 1	0.03	≥ 8	0.25	**0.03**	SYN	**195.97**	SYN mod
**UPV/EHU 17-279**	N.A.	≥ 1	0.06	≥ 8	0.125	**0.04**	SYN	**177.14**	SYN mod
**UPV/EHU 17-280**	N.A.	≥ 1	0.06	≥ 8	0.25	**0.05**	SYN	**192.57**	SYN mod
**UPV/EHU 17-281**	N.A.	≥ 1	0.06	≥ 8	0.125	**0.04**	SYN	**180.55**	SYN mod
**UPV/EHU 17-285**	N.A.	≥ 1	0.06	≥ 8	0.5	**0.06**	SYN	**183.97**	SYN mod
**UPV/EHU 18-029**	N.A.	≥ 1	0.125	≥ 8	1	**0.13**	SYN	**103.43**	SYN mod

S: Susceptible. R: Resistant. N.A: Not Applicable. A: Anidulafungin. T: Tacrolimus. FICI: Fractional Inhibitory Concentration Index. INT: Interpretation. ΣSYN_ANT: total sum of synergic and antagonistic interactions by Bliss model. SYN: Synergy; SYN mod: moderate synergy.

Table S3. *In vitro* interaction between anidulafungin and tacrolimus against *C. glabrata*, by the non-parametric FICI model (Loewe) and the parametric Bliss model (ΣSYN_ANT).

***C. glabrata* isolate**	**R/S**	**Loewe theory**						**Bliss theory**	
		**MIC A_{alone}**	**MIC A_{comb}**	**MIC T_{alone}**	**MIC T_{comb}**	**FICI**	**INT**	**ΣSYN_ ANT**	**INT**
**UPV/EHU 17-013**	R	≥ 1	≤ 0.002	0.125	0.125	**1.00**	IND	**18.37**	SYN weak
**UPV/EHU 17-236**	R	0.5	0.125	≥ 2	0.06	**0.27**	SYN	**27.97**	SYN weak
**UPV/EHU 17-237**	R	≥ 1	≤ 0.002	0.125	0.125	**1.00**	IND	**-26.57**	ANT weak
**UPV/EHU 17-238**	R	≥ 1	1	0.125	0.03	**0.74**	IND	**-10.46**	ANT weak
**UPV/EHU 03-273**	S	0.06	0.03	≥ 2	0.125	**0.50**	SYN	**20.47**	SYN weak
**UPV/EHU 17-077**	S	0.06	0.03	≥ 2	0.03	**0.50**	SYN	**54.56**	SYN weak
**UPV/EHU 17-078**	S	0.06	0.03	≥ 2	0.03	**0.50**	SYN	**51.93**	SYN weak
**UPV/EHU 17-164**	S	0.06	0.03	≥ 2	1	**0.75**	IND	**38.91**	SYN weak
**UPV/EHU 17-173**	S	0.03	0.015	≥ 2	2	**1.00**	IND	**25.25**	SYN weak
**UPV/EHU 17-181**	S	0.06	0.03	≥ 2	0.5	**0.63**	IND	**34.03**	SYN weak
**UPV/EHU 17-242**	S	0.06	0.03	≥ 2	0.06	**0.50**	SYN	**50.88**	SYN weak
**UPV/EHU 17-297**	S	0.06	0.015	≥ 2	1	**0.50**	SYN	**24.36**	SYN weak
**UPV/EHU 22-119**	S	0.06	0.03	≥ 2	0.03	**0.50**	SYN	**-1.26**	ANT weak

S: Susceptible (≤ 0.06 µg/mL); R: Resistant (> 0.06 µg/mL), following EUCAST Clinical breakpoints for anidulafungin [18]. A: Anidulafungin. T: Tacrolimus. FICI: Fractional Inhibitory Concentration Index. INT: Interpretation. ΣSYN_ANT: total sum of synergic and antagonistic interactions by Bliss model. SYN: synergy; IND: indifference; ANT: antagonism.

Table S4. *In vitro* interaction between anidulafungin and tacrolimus against *C. parapsilosis*, by the non-parametric FICI model (Loewe) and the parametric Bliss model (ΣSYN_ANT).

C. parapsilosis isolate	WT/ mut	R/S	Loewe theory						Bliss theory	
			MIC A_{alone}	MIC A_{comb}	MIC T_{alone}	MIC T_{comb}	FICI	INT	ΣSYN_ ANT	INT
**UPV/EHU 17-031**	WT	N.A	≥ 1	≤ 0.002	0.25	0.125	**0.50**	SYN	**24.30**	SYN weak
**UPV/EHU 17-037**	WT	S	1	0.25	0.125	0.06	**0.73**	IND	**45.75**	SYN weak
**UPV/EHU 17-109**	WT	S	0.06	0.03	≥ 2	1	**0.75**	IND	**30.99**	SYN weak
**UPV/EHU 17-112**	WT	N.A	≥ 1	0.5	0.125	0.06	**0.73**	IND	**36.26**	SYN weak
**UPV/EHU 17-113**	WT	N.A	≥ 1	≤ 0.002	0.06	0.03	**0.50**	SYN	**-2.75**	ANT weak
**UPV/EHU 17-114**	WT	S	1	0.25	≥ 2	0.03	**0.26**	SYN	**88.47**	SYN weak
**UPV/EHU 17-250**	WT	N.A	≥ 1	1	0.125	0.03	**0.74**	IND	**5.913**	SYN weak
**UPV/EHU 17-251**	WT	N.A	≥ 1	≤ 0.002	0.5	0.25	**0.50**	SYN	**-5.49**	ANT weak
**UPV/EHU 17-254**	WT	N.A	≥ 1	≤ 0.002	0.125	0.125	**1.00**	IND	**-23.90**	ANT weak
**UPV/EHU 17-311**	WT	N.A	≥ 1	≤ 0.002	0.125	0.125	**1.00**	IND	**-22.39**	ANT weak
**UPV/EHU 17-327**	WT	N.A	≥ 1	≤ 0.002	0.25	0.125	**0.50**	SYN	**39.96**	SYN weak
**UPV/EHU 17-329**	WT	S	1	≤ 0.002	0.125	0.03	**0.24**	SYN	**1.087**	SYN weak
**UPV/EHU 22-120**	WT	N.A	≥ 1	≥ 1	≥ 2	≥ 2	**2.00**	IND	**9.302**	SYN weak
**UPV/EHU 22-121**	*FKS1* mut	S	1	0.25	≥ 2	0.5	**0.38**	SYN	**63.58**	SYN weak
**UPV/EHU 22-122**	*FKS1* mut	N.A	≥ 1	0.5	≥ 2	0.125	**0.28**	SYN	**123.47**	SYN mod
**UPV/EHU 22-123**	*FKS1* mut	N.A	≥ 1	1	0.5	0.06	**0.62**	IND	**63.71**	SYN weak
**UPV/EHU 22-124**	*FKS1* mut	N.A	≥ 1	0.5	0.125	0.03	**0.49**	SYN	**10.32**	SYN weak
**UPV/EHU 22-125**	*FKS1* mut	N.A	≥ 1	0.5	0.25	0.03	**0.37**	SYN	**35.74**	SYN weak
**UPV/EHU 22-126**	*FKS1* mut	N.A	≥ 1	≤ 0.002	0.25	0.125	**0.50**	SYN	**48.29**	SYN weak
**UPV/EHU 22-127**	*FKS1* mut	S	1	0.25	≥ 2	0.03	**0.26**	SYN	**133.69**	SYN mod
**UPV/EHU 22-128**	*FKS1* mut	N.A	≥ 1	0.5	0.25	0.06	**0.49**	SYN	**60.39**	SYN weak
**UPV/EHU 22-129**	*FKS1* mut	S	1	0.004	0.125	0.125	**1.00**	IND	**-6.397**	ANT weak

S: Susceptible (≤ 4 µg/mL); R: Resistant (> 4 µg/mL). following EUCAST Clinical breakpoints for anidulafungin [18]. N.A.: Not Applicable. WT: wild-type. A: Anidulafungin. T: Tacrolimus. FICI: Fractional Inhibitory Concentration Index. INT: Interpretation. ΣSYN_ANT: total sum of synergic and antagonistic interactions by Bliss model. SYN: synergy; IND: indifference; ANT: antagonism; SYN mod: moderate synergy.

Checkerboard assay results

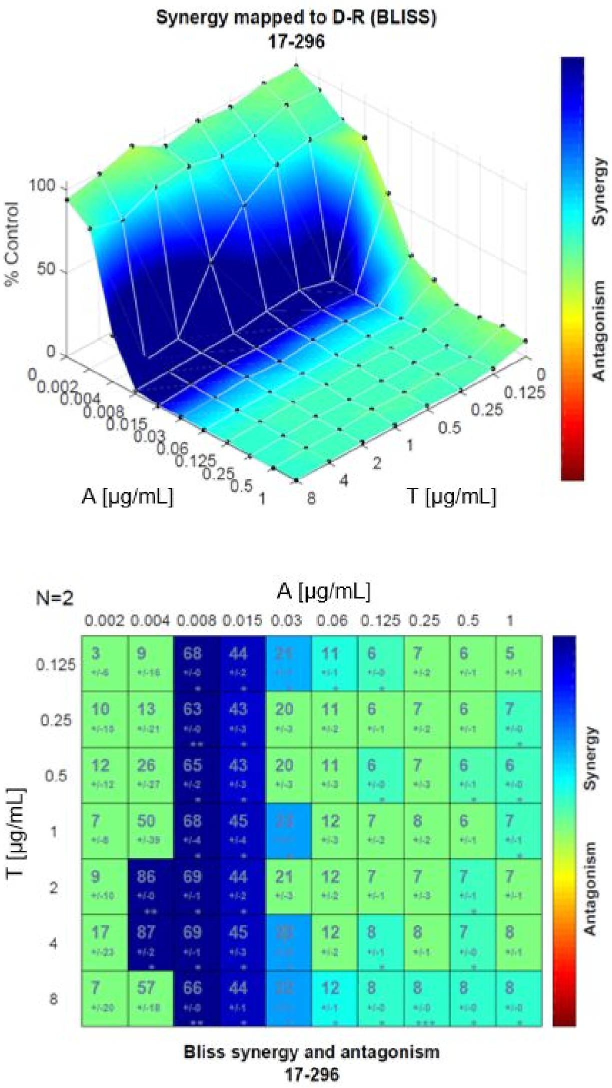

Figure S1. Synergy distribution determined by Bliss interaction model for the combination of anidulafungin and tacrolimus against *C. albicans* UPV/EHU 17-296. Top: Synergy distribution mapped to dose-response surface. Bottom: Matrix synergy plot with synergy scores for each combination.

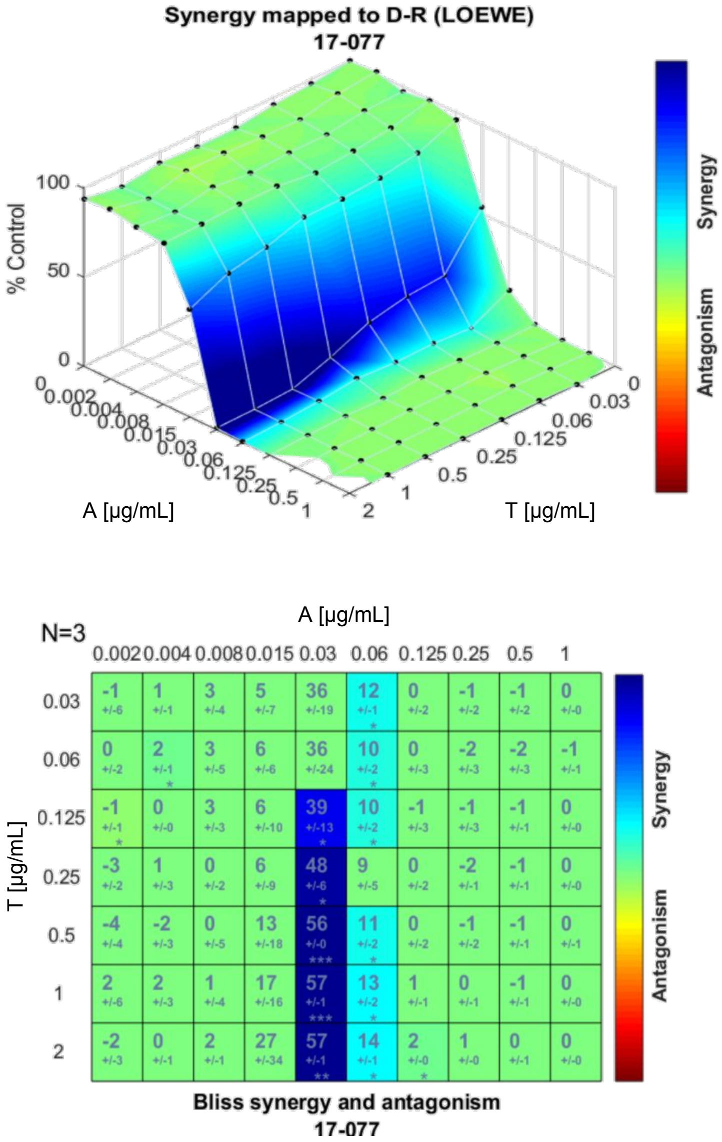

Figure S2. Synergy distribution determined by Bliss interaction model for the combination of anidulafungin and tacrolimus against *C. glabrata* UPV/EHU 17-077. Top: Synergy distribution mapped to dose-response surface. Bottom: Matrix synergy plot with synergy scores for each combination.

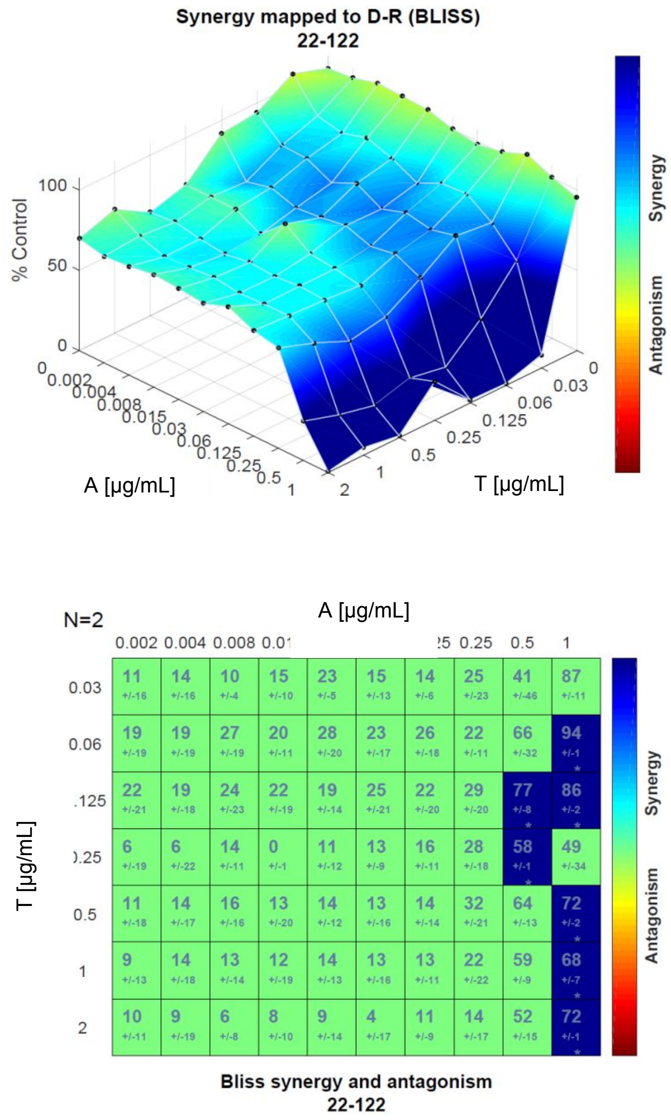

Figure S3. Synergy distribution determined by Bliss interaction model for the combination of anidulafungin and tacrolimus against *C. parapsilosis* UPV/EHU 22-122 with mutation in non-HS regions of *FKS1*. Top: Synergy distribution mapped to dose-response surface. Bottom: Matrix synergy plot with synergy scores for each combination.
